# Recombinant LH priming improves follicular efficiency and pregnancy outcomes in women with diminished ovarian reserve undergoing antagonist IVF stimulation

**DOI:** 10.3389/fendo.2026.1877719

**Published:** 2026-07-24

**Authors:** Diletta Guglielmi, Claudio Maurizio Maria Brigante, Silvana Gippone, Roberta Iemmello, Mariabeatrice Dal Canto, Mario Romano Mignini Renzini

**Affiliations:** Biogenesi, Reproductive Medicine Centre, Monza, Italy

**Keywords:** diminished ovarian reserve, GnRH antagonist, IVF/ICSI, poor ovarian response, recombinant luteinizing hormone

## Abstract

Women with diminished ovarian reserve represent a low-prognosis population in assisted reproduction, in whom improving the efficiency of converting the available antral follicle pool into retrievable oocytes may be clinically relevant. We conducted a retrospective single-center cohort study including 232 IVF/ICSI cycles (232 women) performed between January 2022 and April 2025 in POSEIDON groups 3–4 (anti-Müllerian hormone <1.2 ng/mL), comparing a high-dose antagonist regimen preceded by combined oral contraceptive pretreatment plus recombinant luteinizing hormone priming (150 IU/day for 7 days; LHP, *n* = 93) with a comparable high-dose antagonist regimen without priming (no-LHP, *n* = 139). Both groups received fixed high-dose stimulation with recombinant follicle-stimulating hormone (300 IU/day) plus recombinant luteinizing hormone (150 IU/day). The primary outcome was ongoing pregnancy at ≥12 weeks. Cycle suspension before oocyte retrieval was less frequent with LHP than with no-LHP (1.1% vs. 10.1%). Follicular efficiency also favored LHP, with higher follicular output rate (59.4% vs. 52.8%) and follicle-to-oocyte index (82.6% vs. 70.9%). In fresh transfers, ongoing pregnancy was higher with LHP per started cycle (18.3% vs. 9.4%) and per embryo transfer (30.4% vs. 15.5%), with a similar trend per oocyte retrieval (18.5% vs. 10.4%). In multivariable logistic regression analyses, LH priming remained independently associated with ongoing pregnancy after adjustment for age, body mass index, and anti-Müllerian hormone, with consistent effect estimates across models (odds ratio range 2.20–2.41). Combined first-transfer ongoing pregnancy, defined as the fresh transfer or, when no fresh transfer occurred, the first subsequent frozen embryo transfer derived from the same stimulation cycle, also showed a favorable trend (29.7% vs. 17.0%). On updated follow-up, all pregnancies that had reached the ongoing stage in both groups resulted in live birth. In low-prognosis women, a COC-based recombinant luteinizing hormone priming protocol within a high-dose antagonist framework was associated with improved follicular efficiency, fewer pre-retrieval suspensions, and higher ongoing pregnancy rates in fresh cycles. Prospective studies are warranted to confirm these findings and better define patient selection.

## 1 Introduction

Despite advances in assisted reproductive technology (ART), improving outcomes in women with low ovarian reserve remains a major clinical challenge (1, 2). In patients classified within POSEIDON groups 3–4, the limitation extends beyond oocyte number and involves impaired oocyte competence and suboptimal follicular dynamics (1, 2). In this context, there is increasing interest in strategies aimed not only at improving reproductive outcomes but also at enhancing the efficiency of ovarian stimulation, thereby optimizing the use of the available follicular cohort (1). In this perspective, indices such as the follicular output rate (FORT) and follicle-to-oocyte index (FOI) have been proposed as dynamic markers of ovarian responsiveness, reflecting the efficiency of follicular recruitment and the ability to convert the available follicular pool into retrievable oocytes (3, 4).

According to the two-cell, two-gonadotrophin model, follicular development depends on the coordinated interaction between theca and granulosa cells, mediated by luteinizing hormone (LH) and follicle-stimulating hormone (FSH), respectively (5). LH-driven androgen production in theca cells plays a key role in supporting granulosa cell function and follicular maturation (5). Consequently, modulation of LH activity during the early phases of follicular recruitment may influence intra-ovarian steroidogenesis, improve follicular synchronization, and ultimately affect oocyte competence, which represents a key determinant of subsequent embryo development and implantation potential (5, 6).

Previous clinical evidence, including systematic reviews on recombinant LH use in ART and our prior work investigating LH-based priming strategies targeting theca cell stimulation, has suggested that early exposure to LH may enhance follicular responsiveness in selected low-prognosis patients and may positively influence oocyte competence, thereby potentially improving subsequent embryo development (7–9). These observations support the concept that the timing of LH administration, rather than its mere presence during ovarian stimulation, may represent a critical determinant of both ovarian response and stimulation efficiency (9).

In parallel, oral contraceptive (COC) pretreatment has been widely used for cycle scheduling and synchronization in GnRH antagonist protocols. However, COC pretreatment used as a stand-alone scheduling strategy has been associated with increased gonadotrophin requirements and, in some studies, with less favorable reproductive outcomes, possibly due to excessive suppression of early follicular recruitment (10). Importantly, in those settings, the observed disadvantage is thought to reflect an overly suppressive endocrine milieu not counterbalanced by targeted gonadotrophin support; in our approach, this potential drawback is mitigated by the concurrent administration of recombinant LH during the late phase of COC exposure, aiming to support theca cell activity and maintain an adequate steroidogenic environment at a critical stage of follicular recruitment (10).

Nevertheless, COC pretreatment may offer practical and biological advantages. Compared with GnRH agonist downregulation, it may reduce the incidence of functional ovarian cysts and allow flexible cycle scheduling, which is particularly relevant in clinical practice (11). Moreover, by avoiding GnRH agonist downregulation and operating within an antagonist framework, this approach preserves flexibility for ovulatory triggering, including the option of a GnRH agonist trigger when clinically indicated, rather than being restricted to hCG-only triggering as in long agonist protocols (12).

In this context, combining COC pretreatment with recombinant LH (rLH) priming represents a novel approach aimed at modulating early follicular development. By introducing LH exposure during the late phase of COC administration, this strategy may promote theca cell activity and intra-ovarian androgen availability at a critical stage of follicular recruitment, potentially enhancing granulosa cell responsiveness, improving follicular synchronization, and increasing the efficiency of ovarian stimulation (5, 9).

To date, evidence on LH priming within GnRH antagonist protocols remains limited, particularly in low-prognosis patients, despite broader evidence on recombinant LH supplementation in ART (1, 8, 9). Moreover, the combination of recombinant LH priming with COC pretreatment has not been specifically investigated in this population. To our knowledge, this represents one of the first studies evaluating an LH priming strategy within a COC-based GnRH antagonist protocol in women with low ovarian reserve (POSEIDON groups 3–4).

The present study was therefore designed to evaluate the clinical impact of a COC-based LH priming protocol compared with a standard GnRH antagonist approach without LH priming, with a focus on ongoing pregnancy outcomes and overall stimulation efficiency.

## 2 Materials and methods

**Figure 1:**
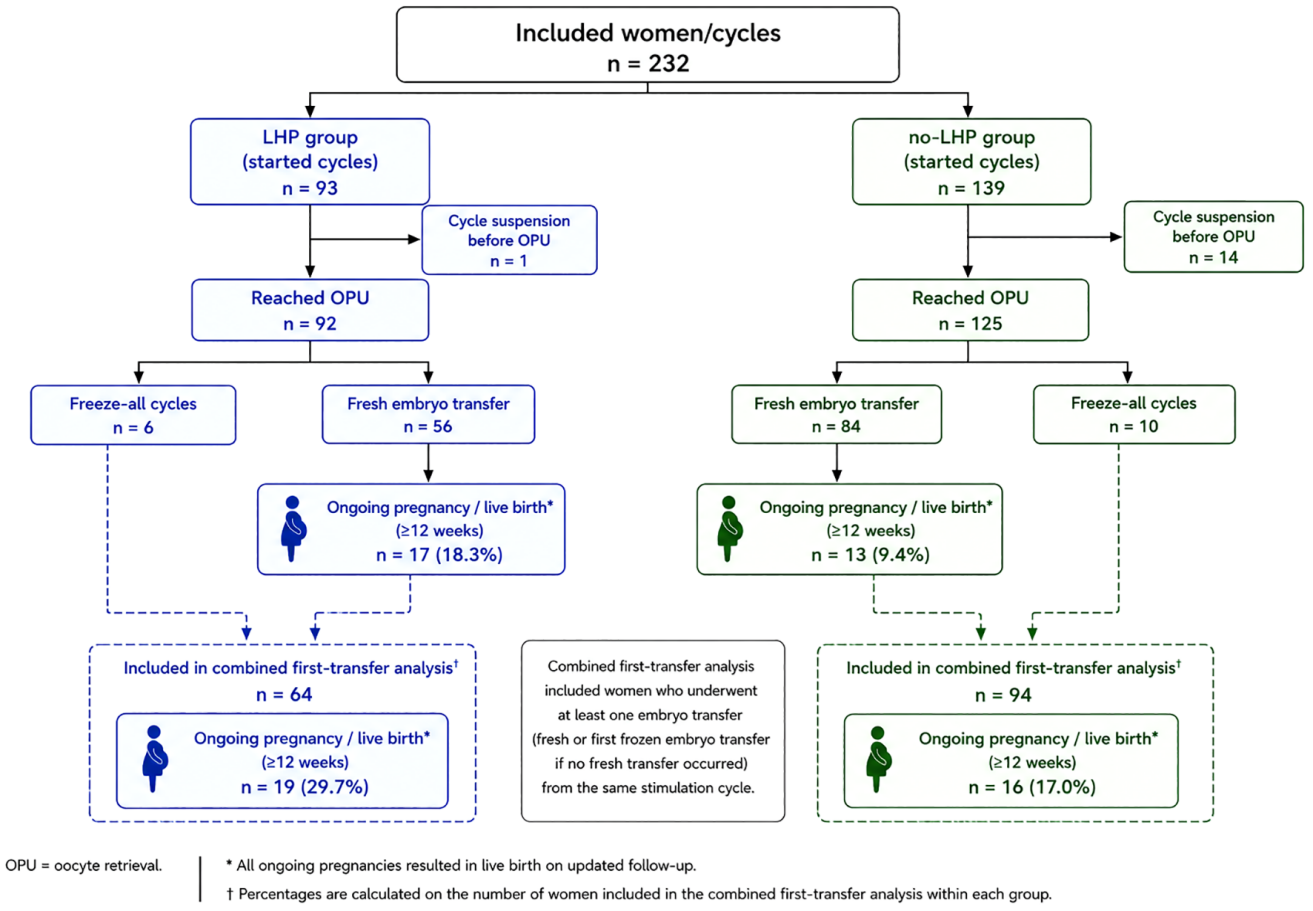
Study flow. Flow diagram of the study population, including cycle suspensions, oocyte retrievals, fresh embryo transfers, freeze-all cycles, and women included in the combined first-transfer analysis. On updated follow-up, all ongoing pregnancies resulted in live birth.

This retrospective, observational, single-center cohort study compared two controlled ovarian stimulation strategies (LHP versus no-LHP) in women undergoing IVF/ICSI at the Biogenesi Reproductive Medicine Centre (Istituti Clinici Zucchi, Monza, Italy). Consecutive cycles performed between January 1, 2022 and April 30, 2025 were screened using the stimulation start date as the index date. Eligible participants were women (≥18 years) undergoing IVF/ICSI with autologous oocytes and classified as low prognosis according to POSEIDON groups 3–4, operationalized as serum anti-Müllerian hormone (AMH) <1.2 ng/mL. The exclusion criteria were as follows: cycles cancelled before initiation of ovarian stimulation, oocyte donation, preimplantation genetic testing, banking cycles without intent of fresh transfer, and FET-only cycles without ovarian stimulation. To reduce intra-individual clustering, only the first cycle per protocol per woman was included (a woman could contribute one LHP and one no-LHP cycle if each was her first exposure to that protocol), resulting in 232 cycles (232 women) analyzed (LHP *n* = 93; no-LHP *n* = 139). Protocol allocation was not randomized and was based on routine clinical practice. Both stimulation strategies were used during the same study period, and treatment selection reflected the overall clinical judgment of the treating physicians rather than predefined allocation criteria. The overall study flow is presented in **Figure 1**.

**Figure 2:**
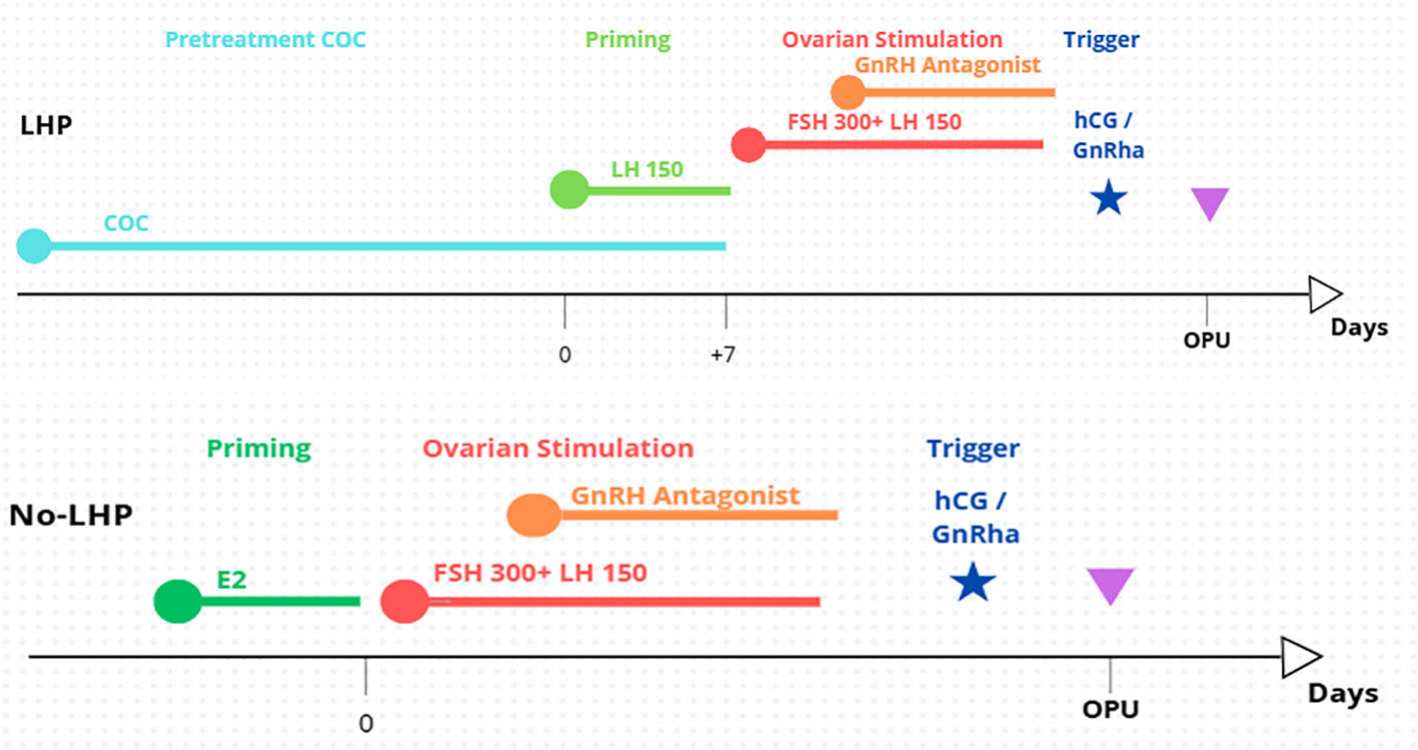
Protocol overview. Schematic representation of the LHP and no-LHP stimulation protocols. LHP included COC pretreatment, followed by rLH priming and GnRH antagonist stimulation, whereas no-LHP included luteal oral estradiol pretreatment without LH priming before GnRH antagonist stimulation.

LHP consisted of combined oral contraceptive (COC) pretreatment for scheduling/synchronization with recombinant LH (rLH) priming (150 IU/day) during the last 7 days of COC, followed by fixed high-dose stimulation with recombinant FSH (rFSH) 300 IU/day plus rLH 150 IU/day; doses were not adjusted during stimulation. No-LHP used luteal-phase oral estradiol pretreatment (4 mg/day) followed by the same fixed high-dose stimulation (rFSH 300 IU/day plus rLH 150 IU/day; FSH/LH 2:1), without LH priming. In both protocols, GnRH antagonist (0.25 mg/day) was introduced flexibly according to follicular development. Follicular growth was monitored by serial transvaginal ultrasound and serum estradiol and progesterone measurements. Final oocyte maturation was triggered with hCG, a GnRH agonist, or a dual trigger according to follicular development and endocrine profile, and transvaginal ultrasound-guided oocyte retrieval was performed 36 h after trigger administration under conscious sedation. Fertilization was performed by IVF or ICSI according to semen parameters, and normal fertilization was defined as two pronuclei (2PN). Embryos were assessed at the cleavage stage (days 2 and 3) and, when extended culture was performed, at blastocyst stage (days 5 and 6). Fresh embryo transfers were performed at cleavage stage (days 2 and 3). Embryos not transferred fresh were cultured to blastocyst stage and cryopreserved by vitrification exclusively at days 5 and 6; frozen embryo transfers in the combined first-transfer ongoing pregnancy analysis used vitrified–warmed blastocysts derived from the same stimulation cycle. Although cycles were initiated with the intent to perform a fresh transfer, a freeze-all strategy was adopted in a subset of cycles when clinically indicated (e.g., premature progesterone elevation or other clinical considerations); such cycles were excluded from fresh outcome analyses but included in combined first-transfer ongoing pregnancy outcome assessment. The two stimulation protocols are schematically presented in **Figure 2**.

The primary outcome was ongoing pregnancy at ≥12 weeks of gestation, defined as a viable intrauterine pregnancy confirmed by ultrasound. Different denominators (started cycles, oocyte retrievals, and embryo transfers) were used to describe treatment progression and outcome distribution across clinically relevant analysis populations.

Secondary outcomes included cycle suspension before oocyte retrieval, clinical pregnancy, implantation, miscarriage, transfer rate, and combined first-transfer ongoing pregnancy. Cycle suspension before oocyte retrieval was defined as discontinuation before planned retrieval due to complete lack of ovarian response (absence of clinically meaningful follicular development despite stimulation) or poor/suboptimal response considered unlikely to lead to oocyte retrieval and/or a viable embryo transfer; decisions were made by treating physicians according to unit standard clinical policy, based on serial ultrasound monitoring (follicle number and growth dynamics) and serum estradiol trends, applied consistently throughout the study period, with counseling documented in the clinical chart. Combined first-transfer ongoing pregnancy was calculated among women who underwent at least one embryo transfer as the outcome of the fresh transfer or, if no fresh transfer occurred, the first subsequent frozen embryo transfer derived from embryos obtained from the same stimulation cycle (each cycle contributed once). Given one cycle per woman, denominators correspond to women. Antral follicle count (AFC) was assessed at baseline transvaginal ultrasound prior to stimulation initiation. Follicular output rate (FORT) was defined as the number of follicles ≥16 mm on trigger day divided by baseline AFC, and follicle-to-oocyte index (FOI) was defined as the number of oocytes retrieved divided by baseline AFC; both were expressed as percentages and compared between groups using *z*-tests for two independent proportions on aggregated counts.

Data were anonymized prior to analysis and analyzed using IBM SPSS Statistics for Windows (IBM Corp., Armonk, NY, USA). Continuous variables are reported as mean ± standard deviation. Between-group comparisons for continuous variables were performed using Student’s *t*-test when parametric assumptions were considered acceptable; otherwise, Mann–Whitney *U*-test was used. Categorical variables are presented as number (percentage) and were compared using Pearson’s chi-square test or Fisher’s exact test, as appropriate. To assess the independent association between LH priming and ongoing pregnancy following fresh embryo transfer, multivariable logistic regression models were constructed. Covariates were selected *a priori* based on clinical relevance and included maternal age, BMI, and AMH. AFC was not included in the multivariable models due to missing or potentially inconsistent measurements in a retrospective setting and because AMH was considered a more robust and consistently available marker of ovarian reserve. Infertility etiology was not included in the multivariable models due to the high prevalence of mixed or overlapping diagnoses and incomplete categorization in the dataset. Results are reported as odds ratios (OR) with 95% confidence intervals (CI). All tests were two-tailed, and *p* < 0.05 was considered statistically significant; *p*-values between 0.05 and 0.10 were interpreted as a statistical trend. No imputation was performed. This study was a retrospective analysis of anonymized clinical data, and no experimental interventions were performed; ethical approval was not required according to local legislation and institutional policies for retrospective analyses of anonymized data. No potentially identifiable patient data are reported. Datasets are available from the corresponding author on reasonable request, in compliance with institutional and legal requirements. No generative AI was used in the preparation of this manuscript.

## 3 Results

The overall study flow is summarized in **Figure 1**.

Baseline characteristics were comparable between groups (**Table 1**). Overall, 232 women (232 cycles) were included: 93 in the LHP group and 139 in the no-LHP group.

**Table 1:** Baseline characteristics (per started cycle).

Variable	LHP (*n* = 93)	no-LHP (*n* = 139)	*P*-value
Age (years), mean ± SD	38.25 ± 3.32	38.44 ± 3.64	0.690
BMI (kg/m²), mean ± SD	23.35 ± 3.59	22.85 ± 3.84	0.320
AMH (ng/mL), mean ± SD	0.67 ± 0.29	0.62 ± 0.30	0.210
Basal FSH (IU/L), mean ± SD	9.79 ± 3.82	10.26 ± 4.57	0.410
POSEIDON group 3, *n* (%)	16 (17.2)	20 (14.4)	0.690^a^
POSEIDON group 4, *n* (%)	77 (82.8)	119 (85.6)	
Primary infertility, *n* (%)	46 (49.5)	69 (49.6)	1.000^b^
Secondary infertility, *n* (%)	47 (50.5)	70 (50.4)	

Values are per started cycle (one cycle per woman).

AMH, anti-Müllerian hormone; BMI, body mass index.

a *p*-value for the overall distribution of POSEIDON groups 3 and 4 between groups.

b *p*-value for the overall distribution of primary versus secondary infertility between groups.

Cycle progression, stimulation characteristics, and follicular efficiency are summarized in **Table 2**. LHP was associated with a significantly lower rate of cycle suspension before oocyte retrieval and higher follicular efficiency indices (FORT and FOI) despite longer stimulation duration and higher gonadotrophin consumption.

**Table 2:** Cycle progression, stimulation characteristics, and follicular efficiency.

Variable	LHP	no-LHP	*p*-value
Cycle progression			
Started cycles, *n*	93	139	–
Cycle suspension before OPU, *n*/*N* (%)	1/93 (1.1)	14/139 (10.1)	0.006
Cycles reaching OPU, *n*/*N* (%)	92/93 (98.9)	125/139 (89.9)	–
Stimulation characteristics^a^			
Stimulation duration (days), mean ± SD	13.40 ± 2.42	10.47 ± 2.23	<0.0001
Total gonadotrophin dose (IU), mean ± SD	4040.2 ± 715.2	3136.8 ± 667.9	<0.0001
Follicular development			
Follicles at baseline ultrasound, mean ± SD	5.70 ± 3.01	5.73 ± 2.50	0.937
Follicles ≥16 mm at last ultrasound^b^, mean ± SD	3.42 ± 2.00	3.37 ± 1.76	0.848
Oocyte yield^c^			
Oocytes retrieved per cycle, mean ± SD	4.71 ± 3.16	4.06 ± 2.83	0.119
MII oocytes per cycle, mean ± SD	2.75 ± 2.16	2.78 ± 2.31	0.922
MII rate, mean ± SD	0.61 ± 0.30	0.67 ± 0.30	0.147
Follicular efficiency^d^			
FORT (%)	59.4	52.8	0.017
FOI (%)	82.6	70.9	<0.0001

OPU, oocyte retrieval; MII, metaphase II oocytes; FORT, follicles ≥16 mm on trigger day divided by AFC; FOI, oocytes retrieved divided by AFC.

a Calculated among cycles reaching OPU.

b Calculated among cycles not suspended before OPU.

c Calculated among cycles reaching OPU.

d FORT and FOI were calculated on aggregated counts and compared using a z-test for two independent proportions.

The laboratory outcomes are summarized in **Table 3**. Among cycles reaching oocyte retrieval, fertilization was performed predominantly by ICSI. Embryological outcomes were broadly comparable between groups, with no significant differences in the number of inseminated/injected oocytes, 2PN zygotes, fertilization rate, or viable day 2 embryos per OPU cycle.

**Table 3:** Laboratory outcomes (cycles reaching OPU).

Variable	LHP	no-LHP	*p*-value
Inseminated/injected oocytes per cycle, mean ± SD	2.60 ± 2.08	2.78 ± 2.30	0.560
2PN zygotes per cycle, mean ± SD	1.67 ± 1.60	1.84 ± 1.74	0.460
Fertilization rate (2PN/oocytes)	0.64	0.66	0.590
Viable day 2 embryos per OPU cycle, mean ± SD	1.68 ± 1.60	2.05 ± 1.72	0.110

2PN, two pronuclei; OPU, oocyte retrieval.

Fresh transfer outcomes are summarized in **Table 4**. Fresh transfer analyses excluded freeze-all cycles, which were included in the combined first-transfer analysis. Fresh embryo transfer and implantation rates were comparable between groups. Clinical pregnancy was numerically higher in the LHP group, whereas ongoing pregnancy was significantly higher with LHP per started cycle and per embryo transfer; the difference per OPU showed the same direction and a borderline trend. No miscarriages occurred in the LHP group, whereas three were observed in the no-LHP group. On updated follow-up, all pregnancies that had reached the ongoing stage in both groups resulted in live birth; therefore, in this cohort, live birth numerically coincided with ongoing pregnancy.

**Table 4:** Fresh transfer outcomes.

Outcome	LHP	no-LHP	*p*-value
Fresh embryo transfer, *n*/*N* (%)	56/92 (60.9)	84/125 (67.2)	0.340
Implantation rate, *n*/*N* (%)	14/81 (17.3)	19/124 (15.3)	0.710
Clinical pregnancy per started cycle, *n*/*N* (%)	18/93 (19.4)	16/139 (11.5)	0.098
Ongoing pregnancy ≥12 weeks per started cycle, *n*/*N* (%)	17/93 (18.3)	13/139 (9.4)	0.047
Ongoing pregnancy ≥12 weeks per OPU, *n*/*N* (%)	17/92 (18.5)	13/125 (10.4)	0.088
Ongoing pregnancy ≥12 weeks per embryo transfer, *n*/*N* (%)	17/56 (30.4)	13/84 (15.5)	0.036
Live birth per started cycle, *n*/*N* (%)	17/93 (18.3)	13/139 (9.4)	0.047
Live birth per OPU, *n*/*N* (%)	17/92 (18.5)	13/125 (10.4)	0.088
Live birth per embryo transfer, *n*/*N* (%)	17/56 (30.4)	13/84 (15.5)	0.036
Miscarriage per clinical pregnancy, *n*/*N* (%)	0/18 (0)	3/16 (18.8)	0.090

All fresh embryo transfers were at cleavage stage (days 2 and 3). On updated follow-up, all pregnancies that had reached the ongoing stage resulted in live birth.

Given the higher ongoing pregnancy rates observed in the LHP group, a multivariable logistic regression analysis was performed to evaluate whether this association was independent of major prognostic factors, with ongoing pregnancy following fresh embryo transfer as the dependent variable (**Table 5**). In the age-adjusted model, LH priming showed a borderline significant association with ongoing pregnancy (OR 2.20, 95% CI 1.00–4.85; *p* = 0.050). After further adjustment for BMI, the association became statistically significant (OR 2.28, 95% CI 1.03–5.05; *p* = 0.041). When adjusting for ovarian reserve using AMH, LH priming remained independently associated with ongoing pregnancy (OR 2.41, 95% CI 1.08–5.39; *p* = 0.032). In the fully adjusted model including age, BMI, and AMH, the association persisted (OR 2.35, 95% CI 1.02–5.44; *p* = 0.045). Maternal age was consistently and inversely associated with ongoing pregnancy across all models, whereas BMI and AMH were not independently associated with the outcome. Overall, the effect of LH priming on ongoing pregnancy was consistent across all model specifications, with odds ratios ranging from 2.20 to 2.41, supporting the robustness of the association.

**Table 5:** Multivariable logistic regression analyses for ongoing pregnancy in fresh-cycle analysis.

Variable	Model 1 (age-adjusted) OR (95% CI)	*p*-value	Model 2 (age + BMI) OR (95% CI)	*p*-value	Model 3 (age + AMH) OR (95% CI)	*p*-value	Model 4 (fully adjusted) OR (95% CI)	*p*-value
LH priming (LHP vs. no-LHP)	2.20 (1.00–4.85)	0.050	2.28 (1.03–5.05)	0.041	2.41 (1.08–5.39)	0.032	2.35 (1.02–5.44)	0.045
Age (per year)	0.87 (0.78–0.97)	0.010	0.86 (0.77–0.96)	0.008	0.85 (0.76–0.95)	0.005	0.85 (0.75–0.96)	0.009
BMI (per kg/m²)	—	—	0.97 (0.90–1.05)	0.450	—	—	0.98 (0.90–1.07)	0.620
AMH (per ng/mL)	—	—	—	—	1.18 (0.98–1.42)	0.080	1.16 (0.96–1.41)	0.110

Data are presented as odds ratios (OR) with 95% confidence intervals (CI) derived from logistic regression models. Model 1 was adjusted for age, model 2 was adjusted for age and BMI, model 3 was adjusted for age and AMH, and model 4 was adjusted for age, BMI and AMH. LH priming corresponds to the V2a protocol. The outcome was ongoing pregnancy following fresh embryo transfer.

Combined first-transfer outcomes are summarized in **Table 6** and were assessed among women who underwent at least one embryo transfer, defined as the fresh transfer or, when no fresh transfer occurred, the first subsequent frozen embryo transfer derived from the same stimulation cycle. This analysis included 64 women in the LHP group and 94 in the no-LHP group. All pregnancy outcomes remained numerically in favor of LHP, with a persistent positive trend for ongoing pregnancy, although without reaching conventional statistical significance. Pregnancy loss was low and comparable between groups.

**Table 6:** Combined first-transfer outcomes.

Outcome	LHP (*n* = 64)	no-LHP (*n* = 94)	*p*-value
Combined first-transfer β-hCG positive, *n*/*N* (%)	26/64 (40.6)	28/94 (29.8)	0.16
Combined first-transfer clinical pregnancy, *n*/*N* (%)	21/64 (32.8)	19/94 (20.2)	0.087
Combined first-transfer ongoing pregnancy ≥12 weeks, *n*/*N* (%)	19/64 (29.7)	16/94 (17.0)	0.080
Pregnancy loss per clinical pregnancy, *n*/*N* (%)	2/21 (9.5)	3/19 (15.8)	0.331

Combined first-transfer analysis includes first frozen transfer only when no fresh transfer was performed; subsequent FETs were not included.

## 4 Discussion

In this retrospective cohort of POSEIDON 3–4 women with diminished ovarian reserve, the COC-based rLH priming protocol evaluated in this study (LHP) was associated with fewer suspensions before oocyte retrieval, higher follicular efficiency (FORT and FOI), and higher ongoing pregnancy rates in fresh transfers compared with a comparable high-dose antagonist regimen without LH priming. Oocyte yield, maturity, and fertilization outcomes were similar between groups. These findings support the concept that the timing and endocrine context of LH exposure may influence follicular competence in low-prognosis women, shifting the focus from gonadotrophin dose escalation toward qualitative endocrine modulation of the theca–granulosa axis (1, 3–6).

The markedly lower rate of cycle suspension before oocyte retrieval in the LHP group represents a clinically relevant finding. In patients with severe diminished ovarian reserve, cycle cancellation before retrieval is a major determinant of reduced success per started cycle. The concomitant increase in follicular efficiency indices (FORT and FOI) suggests that LH priming improves the functional competence of the recruited follicular cohort, enhancing the likelihood of progression to preovulatory stages rather than simply increasing stimulation intensity (1, 3, 4).

Despite similar laboratory outcomes, LHP was associated with higher ongoing pregnancy rates in fresh transfers. This supports the concept that, in low-prognosis patients, improvements in follicular dynamics and endocrine environment may translate into clinically relevant differences not fully captured by conventional embryological parameters. Importantly, both groups received rLH during stimulation, indicating that the observed effect is more likely related to the timing of LH exposure rather than its presence during stimulation per se (6, 8, 9).

The multivariable analysis further strengthens this interpretation. After adjustment for maternal age, BMI, and AMH, LH priming remained independently associated with ongoing pregnancy, with a stable effect size across all models. Maternal age was consistently and inversely associated with outcome, while BMI and AMH were not independently associated in this selected low-prognosis population. The consistency of the effect estimate across different model specifications supports the robustness of the association and reduces the likelihood that the findings are driven by baseline differences or residual confounding.

These findings should be interpreted as a continuation of our previous work, LH Priming Before Ovarian Stimulation in Poor Responders: Effects on Oocyte Recovery and Post ICSI/IVF Pregnancy Rates, in which LH priming was evaluated within a long GnRH agonist protocol and compared with an antagonist-based control regimen. In that study, LH priming was associated with improved ovarian response and pregnancy outcomes, and the association with pregnancy remained significant after multivariable adjustment. However, interpretation was limited by the comparison between two different downregulation frameworks. The present study was specifically designed to address that limitation by reassessing LH priming within a more homogeneous antagonist-based high-dose setting, thereby providing a more focused evaluation of the independent contribution of priming itself (9).

A key design element of the LHP protocol is the use of combined oral contraceptive (COC) pretreatment to achieve the controlled suppression of endogenous gonadotrophins and cohort synchronization within an antagonist framework. This was not adopted as an alternative to long GnRH agonist protocols due to inefficacy but as a strategy to create a reproducible endocrine environment for LH priming while preserving the flexibility of antagonist cycles, including GnRH agonist triggering (10–12).

COC pretreatment in antagonist IVF cycles has been associated with longer stimulation and increased gonadotrophin requirements, findings that are consistent with our results (10). This likely reflects pituitary suppression and delayed early follicular recruitment rather than a direct dose–response relationship between higher gonadotrophin exposure and better outcomes. In this context, the improvement in follicular efficiency and the reduction in cycle cancellation observed with LHP support a qualitative rather than purely quantitative effect of the stimulation strategy.

Importantly, when outcomes were evaluated using the combined first-transfer approach (fresh transfer or first subsequent frozen transfer when no fresh transfer occurred), the positive trend in ongoing pregnancy observed in fresh cycles was maintained. This suggests that the benefit associated with LH priming is not limited to fresh transfer conditions but may extend to the overall reproductive potential of the stimulation cycle (13–15).

The use of COC as a platform for LH priming represents a distinctive aspect of the present protocol. While LH supplementation during ovarian stimulation has been extensively investigated in poor responders and low-prognosis patients, most previous studies evaluated LH administration after the initiation of ovarian stimulation. In contrast, the present study assessed a COC-based recombinant LH priming strategy administered before stimulation onset, with the aim of modulating the endocrine environment during early follicular recruitment. Importantly, the study was conducted in a well-defined POSEIDON 3–4 population and evaluated not only reproductive outcomes but also follicular efficiency indices (FORT and FOI), providing additional insight into potential biological mechanisms underlying the observed clinical findings. Therefore, the novelty of the present study lies less in the use of LH supplementation itself and more in the timing, endocrine context, and mechanistic evaluation of a pre-stimulation LH priming approach (6–9, 16).

This study has limitations inherent to its retrospective design, including non-random protocol allocation and potential residual confounding. As treatment allocation reflected routine clinical decision-making rather than predefined criteria, the possibility of unmeasured selection bias cannot be completely excluded. Although multivariable models were constructed using clinically selected covariates, the number of ongoing pregnancy events was relatively limited. Accordingly, the models were intentionally restricted to a small number of clinically relevant variables to reduce the risk of overfitting, and the fully adjusted model should therefore be interpreted as supportive rather than definitive. Several potentially relevant clinical and embryological variables were not included in the multivariable models, and residual confounding therefore cannot be excluded.

In addition, differences in pretreatment strategy (COC versus luteal estradiol), stimulation duration and gonadotrophin exposure may have contributed to the observed results. Furthermore, because the two groups differed in both pretreatment strategy and LH priming exposure, the specific contribution of LH priming cannot be fully separated from the potential effects of COC-related synchronization and differences in endocrine suppression. Trigger variability and the use of freeze-all in selected cases introduced further heterogeneity. Updated follow-up allowed verification that all pregnancies that had reached the ongoing stage resulted in live birth in both groups. However, live birth was not used as the primary endpoint because the study had originally been designed and analyzed using ongoing pregnancy at ≥12 weeks as the main outcome.

The present findings extend the existing literature on LH supplementation by specifically evaluating a COC-based pre-stimulation LH priming strategy compared with a luteal-phase estradiol-pretreated GnRH antagonist protocol and by demonstrating an association with improved follicular efficiency in a well-defined POSEIDON 3–4 population. The persistence of the observed association after adjustment for major prognostic factors supports the robustness of the findings, although causality cannot be inferred from the retrospective design. Prospective studies are warranted to confirm these findings and to better define the role of COC-based LH priming strategies within individualized stimulation approaches for low-prognosis patients.

## Data Availability

The raw data supporting the conclusions of this article will be made available by the authors, without undue reservation.
